# Improving the Usability and Safety of Digital Health Systems: The Role of Predictive Human-Computer Interaction Modeling

**DOI:** 10.2196/25281

**Published:** 2021-05-27

**Authors:** Chris Paton, Andre W Kushniruk, Elizabeth M Borycki, Mike English, Jim Warren

**Affiliations:** 1 Nuffield Department of Medicine University of Oxford Oxford United Kingdom; 2 Department of Information Science University of Otago Dunedin New Zealand; 3 School of Health Information Science University of Victoria Victoria, BC Canada; 4 School of Computer Science University of Auckland Auckland New Zealand

**Keywords:** digital health, human-centered design, usability, human-computer interaction, predictive modeling

## Abstract

In this paper, we describe techniques for predictive modeling of human-computer interaction (HCI) and discuss how they could be used in the development and evaluation of user interfaces for digital health systems such as electronic health record systems. Predictive HCI modeling has the potential to improve the generalizability of usability evaluations of digital health interventions beyond specific contexts, especially when integrated with models of distributed cognition and higher-level sociotechnical frameworks. Evidence generated from building and testing HCI models of the user interface (UI) components for different types of digital health interventions could be valuable for informing evidence-based UI design guidelines to support the development of safer and more effective UIs for digital health interventions.

## Introduction

User interfaces for digital health systems such as electronic health records (EHRs) or clinical decision support systems should be designed so that clinicians can accomplish tasks efficiently without making errors that could compromise patient safety. Designers of digital health systems should be able to use the best research evidence currently available, drawn from systematic reviews and meta-analyses, to inform their designs. However, the evidence base for designing user interfaces (UIs) of digital health systems has been difficult to establish, as evaluations of UIs are often subjective and difficult to generalize to new clinical contexts [1]. Recent systematic reviews of usability issues with different types of digital health systems (such as computerized physician order entry [2] and electronic medical records [3]) highlight some common issues identified across different studies but also describe the difficulties in generalizing guidance from context-specific evaluations. This evidence is of use to designers but does not offer specific design patterns or quantitatively demonstrate the trade-offs between efficiency and effectiveness that may be involved in different approaches to making designs more usable. Partly due to the weakness of the scientific evidence base, usability guidelines have therefore generally recommended adopting a human-centered design (HCD) approach and the use of expert heuristics to guide the design of interfaces rather than quantitatively validated design patterns.

In this paper, we examine how the use of human-computer interaction (HCI) predictive models can contribute to building a more robust and generalizable evidence base for UI designs for digital health interventions. This evidence base could then be used to advance UI design guidelines and could be incorporated in the human-centered design process to accelerate innovation and improve clinical safety.

HCI modeling was used to develop the first computer mouse [4,5] and the modern window-based graphical UIs in wide use today [6]. As digital health systems become widely (albeit often reluctantly) adopted in health care, HCI modeling could have an important role in ensuring that the systems we use to care for patients are as safe and effective as other medical innovations such as drugs and diagnostics.

To discuss this approach, we provide a summary of the historical origins of predictive HCI modeling, show examples of how it can be used in modern digital health system design, and show how HCI modeling can be integrated into the human-centered design process.

## The Digital Health Design Evidence Gap

**Figure 1:**
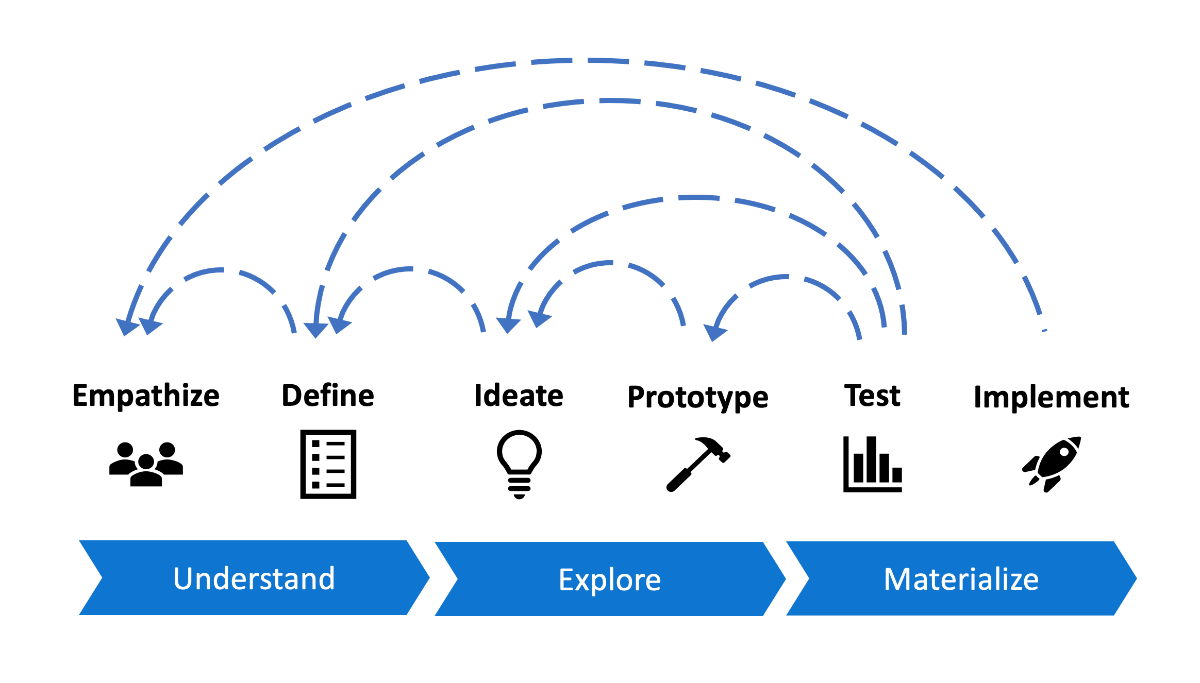
Human-centered design helps designers move from computer code to real-world use. Adapted from Gibbons [7].

The current best practice for designing UIs for digital health systems is to use a human-centered design (HCD) approach such as “design thinking” [7], in which designers and developers move through an iterative and flexible process of understanding, exploring, and materializing the end product (Figure 1). This process is often guided by design heuristics (“rules of thumb”) that include such guidance as keeping the UI simple and aesthetically pleasing and ensuring that help and documentation are readily available (see Textbox 1 for the 10 Nielsen heuristics) [8,9,10]. Iterative design thinking methods attempt to ensure that systems are aligned with users’ behaviors and needs and allow for improvements to be made throughout the course of the design process.

**The 10 Nielsen usability heuristics.**

1. Visibility of system status
2. Match between system and the real world
3. User control and freedom
4. Consistency and standards
5. Error prevention
6. Recognition rather than recall
7. Flexibility and efficiency of use
8. Aesthetic and minimalist design
9. Help users recognize, diagnose, and recover from errors
10. Help and documentation

HCD has been developed to ensure that UIs work well for specific contexts but does not provide the kind of evidence normally expected for medical interventions. HCD should be part of the design process; however, additional methods are needed with a more scientific basis to be confident that digital health UI designs are suitable for use in high-risk settings such as hospitals.

## Predictive Models of HCI

**Figure 2:**
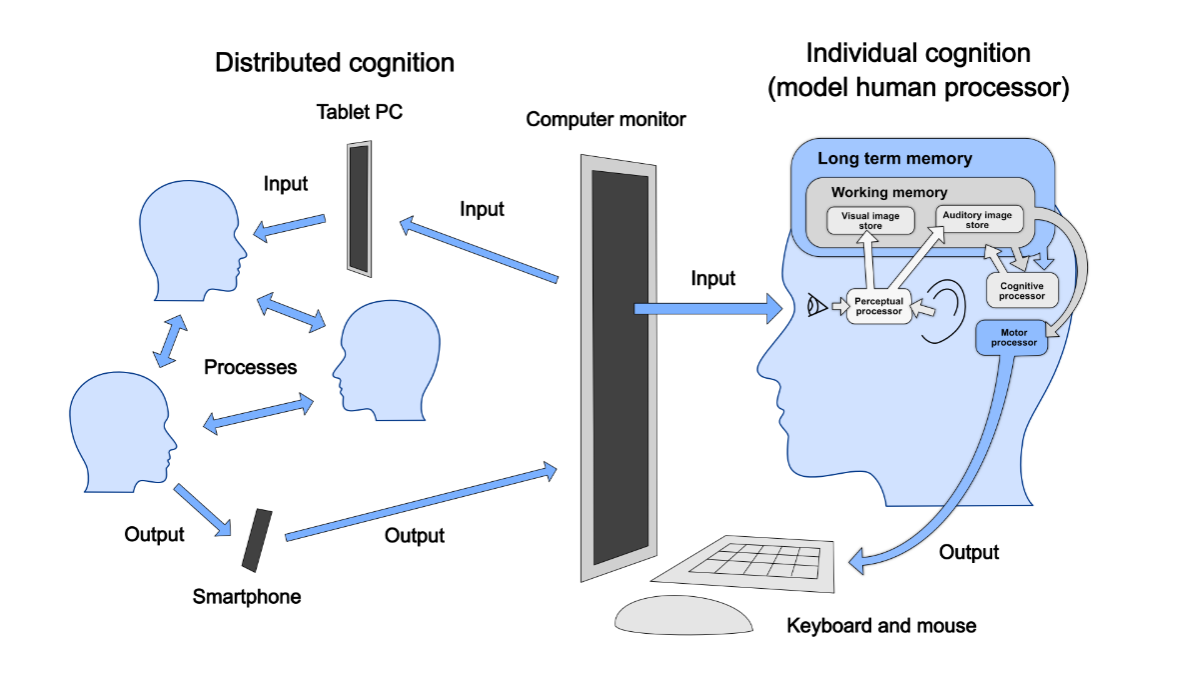
The model human processor: a model of how long it takes to process information (from perception to action) and how we can use the limited “chunks” of information in working memory. Building on the idea of a model human processor is the concept of “distributed cognition,” with multiple humans and devices working together. Adapted from Card et al [11].

Predictive HCI models have the potential to explain how users interact with digital health interventions at the level of individual human cognition. For more than 50 years, empirically derived predictive HCI models have been used for ensuring the safety and usability of information systems (physical and digital) for industrial and commercial applications ranging from avionics to power plant control systems. These models informed the designs of the first desktop computers, with innovations such as the computer mouse and windows-based graphical UIs. The “classic” HCI model was called the “model human processor,” in which the different components of human cognitive systems were modeled and combined with models of the interactions (inputs and outputs) between the human and the computer system (see Figure 2).

There are now several types of HCI predictive models that can be used depending on the context or type of tasks analyzed. For example, the Fitts law is used for mouse pointing [12], the Hick-Hyman law is used for reviewing a sorted list [13,14], and goals operators methods and selection rules (GOMS) [15] is a high-level model that is used to describe the cognitive processes and methods involved in using a computer to achieve specific goals. The keystroke-level model (KLM) is a more specific type of GOMS model that is used for compositions of tasks that fit how an experienced user interacts with the interface [11]. KLM has also recently been updated for touch interfaces [16]. Although individuals will vary in their performance speed, these models give a good indication of the relative effort required to accomplish a task. For example, Warren et al and others [17-19] have used such models for simulation-based evaluation of split-menu designs (placing commonly accessed options at the top of a list) for clinical information systems. These models are primarily aimed at reducing the time needed to complete tasks by eliminating unnecessary clicks and ensuring that UI elements are easy to navigate. In the health care domain, simplifying designs and increasing efficiency is also likely to reduce errors that can cause patient harm, such as choosing incorrect items from unnecessarily long menus or clicking through lengthy screens too quickly [20,21].

## Limitations of HCI Modeling

Although the abovementioned models proved useful and effective in the design of early graphical UIs and input devices, the ways in which teams of people began to use computer systems in the 1990s prompted a move in HCI research communities away from the micro-level interactions to meso-level systems of “distributed cognition” [22] (Figure 2) and “situated action” [23]. These systems included the described cognitive models by attempting to place them within a social context, with human-human interaction playing a role in addition to machine-machine interaction (Figure 2 shows how distributed cognition can be integrated with micro-level HCI modeling). In the health care domain, Borycki and Kushniruk [20,24-26] led the development of an integrative cognitive-sociotechnical model for characterizing user interactions with health care systems at multiple levels.

The field of cognitive science has also moved on since HCI modeling was first proposed. The human cognitive system processes information in a highly complex manner, with dynamic feedback loops that are not included in the ways that HCI models are traditionally described. Therefore, HCI modeling has been more recently viewed as a useful tool for assisting in the development of systems rather than the scientific pursuit originally envisaged by Card, Moran, and Newell in their classic text “The Psychology of Human-Computer Interaction” [15]. However, recent work, such as on the representation of “M” (for “mentally prepare”) in the KLM model, shows that more nuanced and complex models can be created where more fidelity to human cognition is required.

## Example of the “Combined” Layered Approach to Collecting Evidence About HCI

In their study of electronic whiteboards across Denmark, Rasmussen and Kushniruk [27] incorporated the KLM model; however, this work was initially driven by a naturalistic study to locate workflow issues associated with deployment of a new electronic whiteboard across several hospitals across Denmark. The naturalistic study identified areas where optimization might be needed, as evidenced by observation of seemingly inefficient user interactions with the whiteboard (identified from review of the screen recordings of user interactions), and the KLM experimental approach was then used to test hypotheses on applying different changes to create efficiencies.

The research team video recorded 2863 entries from video analysis of live user interactions with the whiteboard and identified potential inefficient sequences from observing and timing the video (eg, the task “add new patient” took an average of 12.3 seconds). They then conducted GOMS-KLM analysis producing the following predictive model (H=move hands; M=mentally prepare; K=tap key or button; P=point):

H+M+P+K+M+P+K+H+M+ (K*10) +H+M+P+K+P+K+H+M+ (K*30)+H+M+P+K+M+P+K+M+P+K+M+P+K+M+P+K+ M+P+K+M+P+K+M+P+K+M+P+K+M+P+K+M+P+K+M+P+K = 54.6 seconds

They then modified the “add new patients” task so that instead of opening new dialog boxes, information could be directly entered into text boxes or from menus, modeled thus:

H+M+P+K+M+P+K+H+M+K+ (K*10) +H+M+P+K+H+M+ (K*30) +H+M+P+K+M+P+K+M+P+K+M+P+K=31.25 seconds

The results of the project led to a reduction in time to task completion of 44.6% and illustrated the benefits of considering HCI at multiple levels (ie, including the use of naturalistic observation and video coding of those data for the use of KLM to predict more optimal user designs for improving inefficient user interaction sequences).

## HCI Model Patterns for Different Types of Digital Health Systems

Digital health applications may have common usability and resultant patient safety issues that can be modeled using predictive HCI approaches as shown above. However, specific types of digital health interventions may also have type-specific UI patterns that, if modeled as a common function of a particular type of system, may make it easier to develop more general models. Using the World Health Organization Digital Health Intervention (DHI) classification system [28], it could be envisaged that each type of system, such as a telemedicine system (DHI 2.4) or health care provider training system (DHI 2.8), would have a common HCI predictive model that takes into account the cognitive processes involved in using that type of system. For example, a training system would include cognitive models of how the system enables the user to learn how to manage a clinical problem, retain the knowledge over time (perhaps by repeatedly “topping up” their knowledge), and recall the information when needed.

## Implications for Developing Guidelines and Standards for Digital Health Systems

Safety issues with large-scale EHR systems have now started to be reported in the literature. Recent work by Ratwani et al [29,30] has highlighted a wide range of usability issues in currently used digital health systems. The analysis by Pacheco et al [31] of the Certified Health IT Product List database of usability attestation, for example, revealed that 3.7% of the products surveyed had a certified capability nonconformity issue that was coded as being associated with possible patient harm. Despite these ongoing issues with clinical usability, current guidance on digital health system development has largely taken a heuristic approach [32], building on and adapting the Nielsen heuristics for health care contexts [3]. Several large-scale projects have been undertaken to develop UI guidelines; however, without establishing an evidence-based approach to guideline development, it has been difficult to maintain or build on these guidelines as technology develops. For example, in the United Kingdom, the National Health Service commissioned Microsoft to develop a “Common User Interface” for EHR systems designed as part of the National Programme for Information Technology; however, this guidance was recently withdrawn without replacement [33]. In the United States, the Department of Health and Human Services created “Research-Based Web Design and Usability Guidelines” [34].

Although these approaches can aid the design of systems that adhere to industrial usability standards, they represent a broad-brush approach that lacks the kind of scientific rigor required by other health care interventions, such as new pharmaceuticals.

Greater consideration and use of predictive models integrated into an HCD approach may be needed to ensure that evidence-based UI design guidelines can be developed over time. The results of modeling-based studies could ensure that systematic reviews and meta-analyses of usability studies generate evidence that is generalizable beyond the specific contexts of the studies.

## Integrating Predictive Modeling With HCD

**Figure 3:**
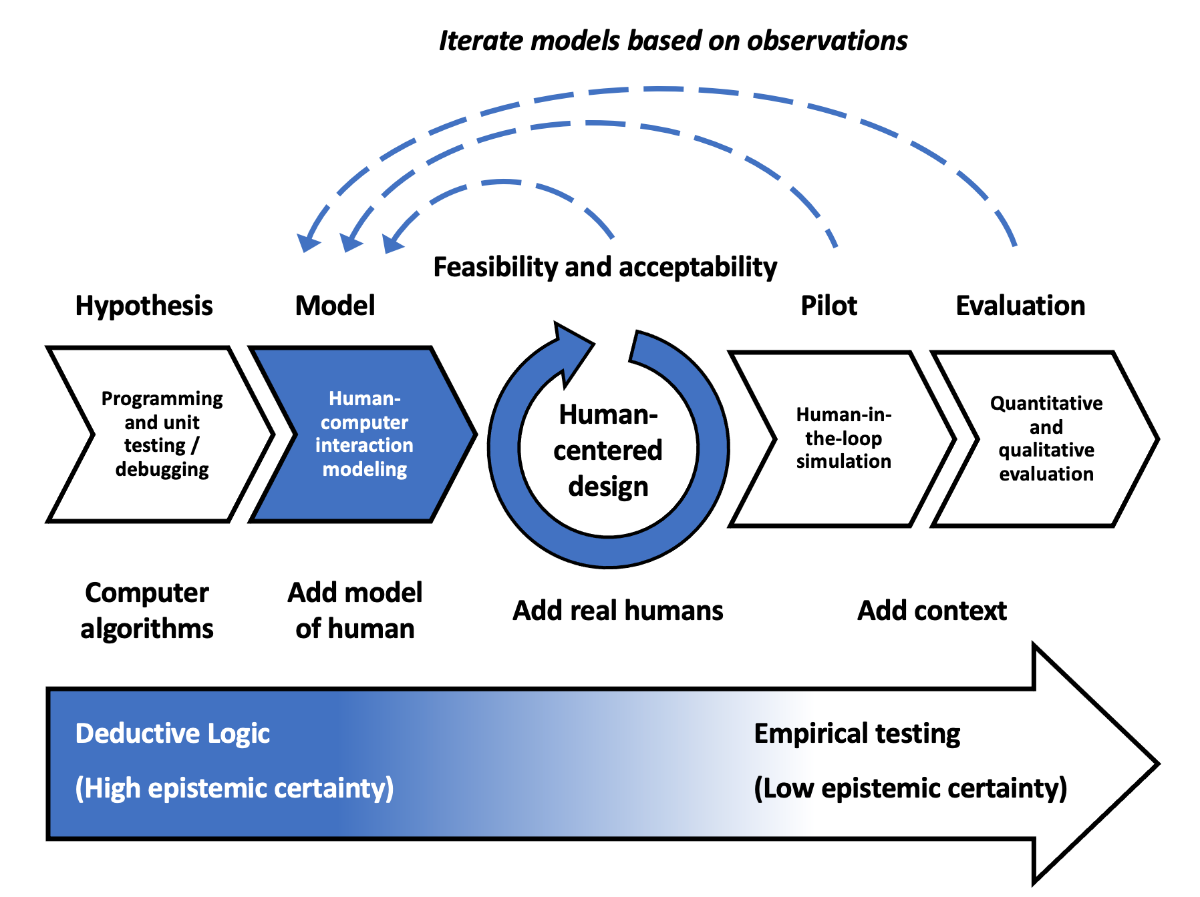
Predictive human-computer interaction modeling could augment the human-centered design process and help us understand how an application achieves real-world effectiveness.

Using predictive models to inform the HCD process could accelerate the design of digital health systems. By having a validated evidence base of UI designs to draw on, designers could eliminate a large number of potential designs that might meet basic usability heuristics or that could be appealing to early testers but that could be shown through predictive modeling to have poor usability. Figure 3 shows how HCI modeling could fit into the process of designing the UI for a digital health application. The design process moves from implementing the computer algorithms needed for the software to function (developed using deductive logic with a high level of epistemic certainty) to modeling how users would interact with the UI of the system using HCI cognitive models. Once designs that show poor results with modeling are weeded out, the project will then enter a human-centered design phase in which the system is trialed with real users (for example, nurses and physicians who will use a digital health system on the wards) and repeatedly iterated until the software is sufficiently acceptable to pilot. At this stage, human-in-the-loop simulations can be conducted as the system is piloted. Finally, more formal quantitative and qualitative evaluations in clinical contexts can provide higher-level empirical evidence (albeit with lower epistemic certainty than with in-silico HCI modeling). At each stage, in keeping with the design thinking approach, the development team can move back to modeling and HCD to improve the design if needed. In addition to showing whether a particular application works, real-world evaluations based on HCI models will show which models work in the real world, building the evidence base for future design guidelines.

## Conclusion

UIs for digital health applications are currently designed using techniques developed for commercial software applications based on human-centered design processes and heuristics. Predictive HCI modeling of applications may help improve the design process and allow for more scientific progress toward safer and more effective digital health systems. We have described in this paper how predictive HCI modeling has developed from individual cognitive modeling to distributed cognitive models and provided examples of how these models can be integrated into sociotechnical modeling approaches. Although predictive HCI modeling has fallen out of favor in recent years, as the demand for more evidence of the safety and effectiveness of digital health systems increases, it is worth re-evaluating whether HCI modeling can contribute to the science of evidence-based digital health system design. Future research on the integration of predictive modeling with usability and software engineering approaches (such as usability testing and human-in-the-loop simulations) is both needed and warranted.

## Abbreviations

DHI: digital health intervention
EHR: electronic health record
GOMS: goals operators methods and selection rules
HCD: human-centered design
HCI: human-computer interaction
KLM: keystroke-level model
UI: user interface
